# Evaluation Of Mycotoxin Content In Soybean (Glycine max L.) Grown In Rwanda

**DOI:** 10.18697/ajfand.83.17710

**Published:** 2018-12

**Authors:** M Niyibituronsa, AN Onyango, S Gaidashova, SM Imathiu, M Uwizerwa, I Wanjuki, F Nganga, JC Muhutu, J Birungi, S Ghimire, K Raes, M De Boevre, S De Saeger, J Harvey

**Affiliations:** 1 Rwanda Agriculture Board, Rwanda, P.O. BOX 5016 Kigali, Rwanda; 2 Jomo Kenyatta University of Agriculture and Technology, Kenya, P.O. BOX 62000 (00200) Nairobi Kenya; 3 Biosciences eastern and central Africa-International Livestock Research Institute Hub, Kenya, Nairobi, 00100, Kenya; 4 Department of Food Technology, Safety and Health, Ghent University – Campus Kortrijk, Belgium, 8500 Kortrijk, Belgium; 5 Department of Bioanalysis, Ghent University, Belgium, 9000 Gent, Belgium; 6 Feed the Future Innovation Lab for the Reduction of Post-Harvest Loss, and Department of Plant Pathology, Kansas State University, Manhattan, KS66506, USA

**Keywords:** soybean, safety, mould, aflatoxin, mycotoxins, sterigmatocystine, ELISA, LC-MS/MS, Rwanda

## Abstract

Soybean is a critical food and nutritional security crop in Rwanda. Promoted by the Rwandan National Agricultural Research System for both adults and as an infant weaning food, soybean is grown by approximately 40% of households. Soybean may be susceptible to the growth of mycotoxin-producing moulds; however, data has been contradictory. Mycotoxin contamination is a food and feed safety issue for grains and other field crops. This study aimed to determine the extent of mycotoxin contamination in soybean, and to assess people’s awareness on mycotoxins. A farm-level survey was conducted in 2015 within three agro-ecological zones of Rwanda suitable for soybean production. Soybean samples were collected from farmers (n=300) who also completed questionnaires about pre-and post-harvest farm practices, and aflatoxin awareness. The concentration of total aflatoxin in individual soybean samples was tested by enzymelinked immunosorbent assay (ELISA) using a commercially-available kit. Other mycotoxins were analyzed using liquid chromatography-mass spectrometry (LCMS/MS) on 10 selected sub samples. Only 7.3% of the respondents were aware of aflatoxin contamination in foods, but farmers observed good postharvest practices including harvesting the crop when the pods were dry. Using enzyme-linked immunosorbent assay (ELISA), only one sample had a concentration (11 µg/kg) above the most stringent EU maximum permitted limit of 4 µg/kg. Multi-mycotoxins liquid chromatography-mass spectrometry (LC-MS/MS) results confirmed that soybeans had low or undetectable contamination; only one sample contained 13µg/kg of sterigmatocystine. The soybean samples from Rwanda obtained acceptably low mycotoxin levels. Taken together with other studies that showed that soybean is less contaminated by mycotoxins, these results demonstrate that soybean can be promoted as a nutritious and safe food. However, there is a general need for educating farmers on mycotoxin contamination in food and feed to ensure better standards are adhered to safeguard the health of the consumers regarding these fungal secondary metabolites.

**Figure:**
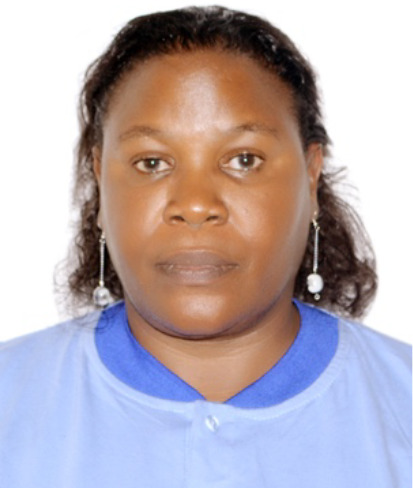
Niyibituronsa Marguerite

## INTRODUCTION

Soybeans (Glycine max L.) are legumes originating from China before 2500BC that were utilized as source of food. The western world discovered soybeans in the 19^th^ century as a source of oil and protein for the human diet [1]. The USA is the leading country with a soybean production of 106.93 million metric tons (MT) in 2016 [2]. In Africa 1.4 million MT were produced from 1.2 million ha [3]. The soybean production in Rwanda was estimated at 57,089MT per year. The area covered in 2012 was 42,160 ha and this ranks Rwanda the 6^th^ African country on soybean area covered after Zimbabwe, Malawi, Uganda, South Africa and Nigeria [4]. In Rwanda the importance of soybean has been recognized as a food and nutritional security crop. This crop has been chosen to increase production in the Crop Intensification Program – CIP [5]. In this regard, there is a need to verify the safety of soybeans produced in Rwanda.

Mycotoxin contamination is a food and feed safety issue, certainly for grains and other staple crops. The complete eradication of mycotoxins is difficult as they are formed both pre- and post-harvest under natural environmental conditions [6]. The mycotoxins of most importance are aflatoxins (AF), fumonisins (FB), ochratoxin (OTA), deoxynivalenol (DON) and zearalenone (ZEN). Aflatoxins are secondary metabolites of *Aspergillus flavus*, *A. parasiticus* and *A. nomius* [7-9], and contaminate a variety of commodities such as cereals, nuts, dried fruit, spices, oil seeds, dried peas, beans and fruits [10].

Aflatoxins and FB have been shown to cause liver damage and cancer [11, 12]. The contribution of AF to hepatocellular damage is estimated to be between 4.6% and 28.2% in the regions with the highest aflatoxin exposure: Sub-Saharan Africa, Southeast Asia, and China [13, 14]. Aflatoxins consumption causes other health problems like acute aflatoxicosis, immune deficiency, and malnutrition-related disorders such as stunting depending on the exposure [12, 13]. Periodic episodes of aflatoxicosis have been reported in the literature and in the media, including many in East Africa. Aflatoxins outbreaks on humans have been reported in many countries for example in India and Kenya [15]. In Kenya, 317 casualties were reported in 2004 [13]. In 2016, 14 deaths from acute aflatoxin poisoning were reported in Tanzania, in the popular media [16]. Although AF accumulate in food commodities stored under conditions that promote fungal growth post-harvest, the initial contamination can start pre-harvest after crop maturity, particularly for certain crops, and under warm and high humidity/wet conditions [9, 17, 18]. Therefore, awareness of and capacity to implement good agricultural practices and good manufacturing practices is one of the strategies proposed to lower health risks associated with mycotoxins in foods [11].

The European Commission has set maximum levels for 11 mycotoxins in foods [19]; however, many common mycotoxins, originating from several fungal genera, do not have maximum levels. Although maximum levels have been set for individual mycotoxins, mixtures of mycotoxins may have additive and/or synergistic effects [20, 21]. Therefore, it is important to determine all mycotoxins in any given food sample. Some studies showed that soybean is susceptible to the growth of moulds that produce mycotoxins such as AF and trichothecenes [6, 22]. However, data has been contradictory on the growth of *A. flavus* and AF production on soybeans [6,23]. Other studies have reported that soybean is less susceptible to mycotoxin contamination than other food and feedstuffs [24, 25]. While no data has been published on mycotoxins contamination in soybeans in Rwanda, it is important to investigate the mycotoxin concentration in that commodity commonly consumed by many Rwandese people including as a weaning food for infants. Soybean products are perceived to be healthy and nutritious foods, and contribute to the food security in the country. Assessing people’s awareness on mycotoxins is key. The provision of information on workable mitigation strategies will enhance the accessibility of safe food and feed in Rwanda.

## MATERIALS AND METHODS

### Sampling

**Figure 1:**
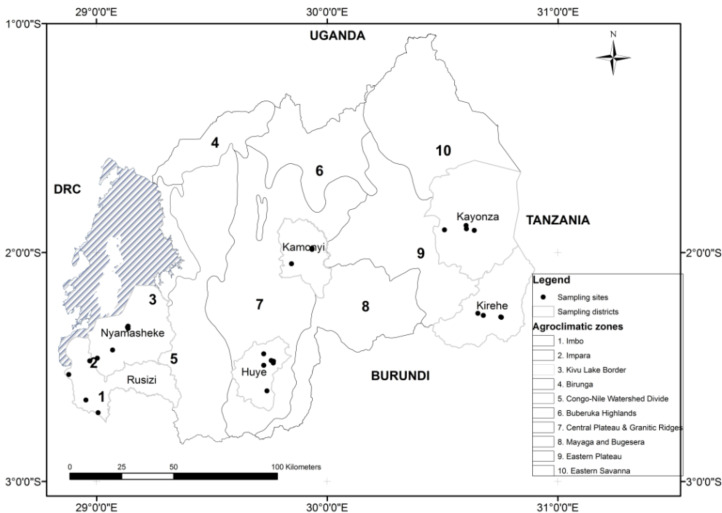
Sampling sites in agro-ecological zones favourable for soybean growth in Rwanda

A multi-stage method (from a country level, per province level up to district level) was used to randomly select two districts per agro-ecological zone with soybean production, namely Kirehe (average altitude 1521 m) & Kayonza (1431 m) (East-Rwanda), Huye (1704 m) & Kamonyi (1662 m) (South-Rwanda), and Nyamasheke (1677 m) and Rusizi (1501 m) (South-West-Rwanda) (**Fig. 1**) [26]. Within these districts, three soybeanproduction sectors were purposively selected to collect the samples from households, markets and the Rwanda Agriculture Board (RAB) stores in August 2015. Sampling was performed according to the Whitaker guideline for sampling food for mycotoxins analysis [27]. Briefly, the grains were taken using a cup from the upper, middle and lower part of the sack used for storage, and kept in a paper bag before transfer to the RAB Rubona cold room (+4ºC). Soybean samples (n=300) of 1kg each were bought from farmers (n=300) who also responded to questions on pre-and post-harvest farm practices, and aflatoxin awareness.

The sample size was calculated according to the Dhulkhed formula [28]

$$n=\frac{{\left(Z_{1/2\alpha }+Z_{\left(1-1/2\beta \right)}\right)}^{2}p(1-p)}{d^{2}}=\frac{\left(1.96+1.282{)}^{2}(0.4)(0.6\right)}{0.1^{2}}=252$$ (equation 1)

The proportion of households who grow soybean was estimated at 40% [29] and the type I error probability (alpha 0.05 giving 1.96) for estimating proportions with probability (power) of 0.90 (1.282) [28, 30]. Samples were increased to 300 in case of loss during transport.

Each bag was labelled with information regarding its collection date, provenance, variety, planting and harvesting time, drying and storage duration before selling/consumption. Other information collected included: equipment used for storage, type of pesticides used and global positioning system (GPS) coordinates. Awareness on aflatoxin contamination was investigated in the local language Kinyarwanda, starting by the knowledge of mould (“uruhumbu”) that can produce toxins (“uburozi”).

One hundred grams from each of the 300 soybean samples was put in a zip-lock bag, and packed in a box (30kg), which was transferred to the Biosciences eastern and central Africa-International Livestock Research Institute (BecA-ILRI) Hub (Nairobi, Kenya) for AF analysis by enzyme-linked immunosorbent assay ELISA. The remaining part was homogenized per district, and subsamples (n=10) from three randomly selected districts in the agro ecological zones (East: Kirehe 2 subsamples, West: Nyamasheke 2, and South: Kamonyi 1 and 5 RAB varieties subsamples) were analysed for multiple mycotoxins using LC-MS/MS (Ghent University, Belgium). Samples were kept in the cold room (4°C). As most surveys have been focusing on aflatoxin only, LC-MS was used to investigate possible occurrence of other mycotoxins in soybeans.

### Sample preparation

Samples were removed from the cold room and put at room temperature for one hour. For aflatoxin analysis, a 50 g sub-sample was taken and ground using a coffee grinder to obtain a fine powder (< 5mm sieve) to facilitate extraction.

For multi-mycotoxins analysis, an IKA® M20 universal mill (Staufen, Germany) was used to grind samples (200g per sample). The grinder was cleaned before milling to avoid cross-contamination. The ground sample was transferred in a zip-lock bag and stored in the cold room.

### Materials and reagents

Total AF assay enzyme-linked immunosorbent assay (ELISA, Cat. No. 981AFL01LM96, Helica Biosystems Inc, Santa Ana, CA 92704, USA) were used. A plate reader was used to measure optical density per manufacturer instructions [31].

For LC-MS analysis, all used reagents were of analytical grade. These include acetic acid, Merck, methanol absolute LC/MS (for mobile phase), Biosolve, n-hexane BDH HiperSolv CHROMANORM, Prolabo, VWR, acetonitrile HPLC-R and mycotoxin standards. ochratoxin A (OTA) (10 µg/mL), aflatoxin mix (AF-mix: AFB1, AFB2, AFG1, AFG2) (20 µg/mL),deoxynivalenol (DON) (100 µg/mL), zearalenone (ZEN) (100 µg/mL), fumonisin mix (FB-mix: FB1, FB2) (50 µg/mL), nivalenol (NIV) (100 µg/mL), neosolaniol (NEO) (100 µg/mL), deepoxy-deoxynivalenol (DOM, 50 µg/mL), T-2 toxin (100 µg/mL), HT-2toxin (100 µg/mL), 3-acetyldeoxynivalenol (3-ADON) (100 µg/mL), 15- acetyldeoxynivalenol (15-ADON) (100 µg/mL), diacetoxyscirpenol (DAS) (100 µg/mL), fusarenon X (FUS-X) (100 µg/mL) and sterigmatocystine (STERIG) (50 µg/mL) were obtained from Biopure, Romerlabs (Darmstadt, Germany). Roquefortin C (ROQ-C) (1 mg) was purchased from Enzo Life Sciences (Axxora Platform, New York, USA), while zearalanone (ZAN, 1 mg), alternariol (AOH) (0.1mg) and alternariol monomethylether (AME, 5 mg) were obtained from Sigma (Brussels, Belgium). Fumonosin B3 (FB3) (1 mg) was purchased from Medical Research Council (Capetown, South Africa).

### Determination of aflatoxin levels in soybean flour samples by ELISA

Enzyme-linked immunosorbent assay was used to measure total AF levels in soybean flour. Ground soybean flour samples (5.0 g) were weighed into 50 mL Falcon tubes. The aflatoxin extraction was done by adding 25 mL of acetonitrile/water (80/20, v/v) in each tube. The tubes were capped, put into an orbital shaker, and shaken at 6 relative centrifugal forces (RCF) at 25°C for 5 minutes. The samples were then left to stand for 15 minutes to allow solids to settle. An aliquot of 100 µL was diluted in 900 µL of reconstituted wash buffer in 2.0 mL tubes, and vortexed for 5 seconds. The ELISA kit consisted of a 96-wells micro titer plate coated with antibody where aflatoxin will bind if present. The analysis was done following the manufacturer’s instructions. A random sample was analysed twice for each plate to measure the accuracy of the data generated. The optical density of the samples and standards were read on a micro titer plate reader at a 450nm wave length filter setting. The computer recorded the optical density (OD) of each micro well, and the concentrations of AF were calculated (µg/kg) from the logit regression equation generated in the standard ODs with r^2^>0.98 for quality data. The limit of detection (LOD) was calculated for each plate in the matrix of standards at 0.02, 0.05, 0.1, 0.2 and 0.4µg/kg. The recovery of 5µg/kg spiked soybean samples (n=3) extracted with acetonitrile/water (80/20, v/v) was 4.6µg/kg (92%) in four independent experiments[31].

### Determination of multi-mycotoxins levels in soybean flour samples by LC-MS/MS

A quantitative LC-MS/MS method was used to determine the levels of multiple mycotoxins in ground soybean samples. An analytical balance (Sartorius, Goettingen, Germany) was used to weigh 5.00 g (± 0.05 g) of sample in an extraction tube of 50 mL. A blank soybean sample was used, and four spikes for the calibration curve were prepared. Internal standards were added, namely 100 µL of ZAN and 25 µL of DOM. Working solutions were added to the spiked samples (0.5 X µg/kg, X µg/kg, 1.5 X µg/kg, 2 X µg/kg), where X was equal to the cut-off level of the mycotoxin. Extraction was done by adding 20 mL of the extraction solvent acetonitrile/water/acetic acid (79/20/1, v/v/v) to the samples. The extraction tubes were wrapped in aluminium foil, and agitated with the shaker for 1 hour, then centrifuged for 15 minutes at 3300 relative centrifugal force (RCF). The supernatants were purified on a C18 column (500 mg/6 mL, Grace, Alltech, Columbia, USA) installed on a vacuum elution manifold with volumetric flasks to collect the eluent which was defatted. From the defatted extract, two ways were followed. First, 12.5ml was added into 27.5ml of acetonitrile/acetic acid (99/1, v/v) and cleaned up through a MultiSep^®^226-column (Romer Labs, Tulln, Austria). Second, 5ml of the remaining solution was filtered through a folded glass filter on a plastic tube of 10ml. Two ml was added to the cleaned eluent (to avoid loss of some compounds like fumonisins that could remain in the column) and evaporated until complete dryness at 40°C under a gentle nitrogen flow. The mobile phase solution (150 µL) was added to dissolve the residue. The filtrate was transferred into a vial for LC-MS/MS analysis. Mycotoxin analysis was performed on an Acquity HPLC-Quattro Premier (Waters, Milford, USA). Four identification criteria were considered to confirm a mycotoxin identified by LC-MS/MS that is two selected fragment ions, a signal to noise ratio >3, a relative retention time of ±2.5% and a relative peak area of ±25%. More details on the used LC-MS/MS are reported in Monbaliu *et al.* [32] and other studies which show that clean up and defatting are key to eliminate interfering compounds [32, 33, 34].

### Quality control and data validation

A re-injection of the cut-off (middle standard of calibration curve) was done at the end of a series analyzed on the LC-MS to check recovery of the LC-MS. Furthermore, the purification and analysis of a blank sample spiked at cut-off level was done to check the recovery of the complete analysis. Two wheat sample controls were used during analysis to evaluate the precision of the data. In-house validation data are detailed in Chilaka [35].

### Data analysis

Data interpretation and analysis were executed using SPSS 16.0 and Excel. Findings could not be tested for statistical significance by ANOVA due to the low percentage of positive samples.

## RESULTS AND DISCUSSION

### Soybean pre- and post-harvest handling

The drying duration of soybean samples by farmers was between one and eight days. Fifty-eight percent of farmers dried their soybeans for 5 days (mean, 4.2±1.5days). The storage duration was between 1 and 4 months, where only 2 samples were stored for 12 months. The storage devices were polyethylene sacks (99%, n=298) and plastic containers (1%, n=2) (**Table 1**). Only 7.7% of the farmers (n=23) used pesticides, normally spermetrine purchased in Agro-vet shops. [Table 1]

**Table 1:** Samples pre and post-harvest handling

		Number of samples (n)	Percentage (%)
	Total	300	100.0
**Source**	Household	138	46.0
	Market	140	46.7
	RAB	22	7.3
**Planting time and harvesting time**	03/2014-07/2014	2	0.7
	09/2014-03/2015	25	8.3
	10/2014-03/2015	20	6.7
	02/2015-06/2015	245	81.7
	04/2015-07/2015	8	2.7
**Drying duration (days)**	1	33	11.0
	2	24	8.0
	3	16	5.3
	4	41	13.7
	5	174	58.0
	6	1	0.3
	7	10	3.3
	8	1	0.3
**Storage duration (in months)**	1	251	83.7
	2	1	0.3
	3	1	0.3
	4	45	15.0
	12	2	0.7

**Table 2:** Extent of Rwandan soybean contamination by aflatoxins

Categorized soybean aflatoxin measurements	Number of samples	Agro-ecological zone	District
**Below LOD (undetectable) <1µg/kg**	291		
**Positive, below maximum allowable limit (µg/kg)**			
1.00	1	South	Kamonyi
1.00	1	East	Kirehe
1.00	1	East	Kayonza
1.10	1	East	Kayonza
1.10	1	West	Nyamasheke
1.20	1	East	Kirehe
1.40	1	East	Kayonza
1.60	1	East	Kirehe
**Positive, above maximum limit (¼g/kg)**			
11.2	1	East	Kirehe

### Aflatoxin awareness

Only 7.3% (n=22) of the respondents were aware on the subject of AF and the related human health impact. These persons were educated staff members of RAB stores. More than 90% (92.7%) of the respondents did not know the term “aflatoxin” and were not aware of the problem of toxins, even not in their local language Kinyarwanda. These findings clearly have shown that there is a need to create awareness on AF among the Rwandese by training to prevent food colonization by mycotoxigenic fungi [36]. Ignorance on the understanding of AF, and conditions in which producing fungal species grow, could lead to acute and chronic toxicity [37], and subsequent AF outbreaks [13, 38]. Thus, it is important to train farmers on mycotoxins contamination in food and feed to ensure better standards are adhered to safeguard the health of the consumers regarding these fungal secondary metabolites.

### Total aflatoxins in soybeans

The quantification of AF was obtained from a logit-log standard curve and the concentrations were adjusted according to a dilution factor of 50. The LOD was 1 µg/kg, the correlation factor (R²) was 0.98. The AF incidence found in soybean in this study was low as shown in Table 2.

Most of the positive samples were collected in the Eastern zone of Rwanda, which is known to have a hot and dry climate which are favourable conditions for *A. flavus* [17, 18, 39].The least contaminated sample above the LOD contained 1 µg/kg AF, and the highest contaminated had 11.2µg/kg from the Eastern region. In the EU, Current maximum levels for total aflatoxins in groundnuts, cereals and dried fruits (AFB1, AFB2, AFG1 and AFG2) are 4 µg/kg (EC 2006). In the US, the maximum limit for grain and grain products is 20 µg/kg [40]. The low levels of AF in the samples are consistent with previous reports that soybeans are not a good substrate for the production of AF even when they are contaminated with *A. flavus* [41, 42]. However, good post-harvest handling like drying duration (on a polyethylene sheet) and grain storage in polyethylene sacks may have contributed to the obtained good quality. Other studies showed higher levels of contamination, such as the study done by Kaaya [43] in Uganda (range, 0 µg/kg – 40 µg/kg) where drying was done on the bare ground. The study done by Dharmaputra [44] in Indonesian soybean meal revealed no contamination in soybean grain, however, a study on the contamination level of AF in some cereals and beans of Pakistan showed that 15% of soybean samples were contaminated [45]. In the present study, it was found that Rwandan farmers harvested when pods were dry, and further dried the beans for an average of 5 days. This facilitates removing the beans without breaking, as broken grains are easily accessible by *A. flavus* [46]. The storage was done in polyethylene sacks after drying for 5 days on plastic sheeting in the sun to reduce the moisture content to 13%. A higher moisture level would contribute to *A. flavus* invasion, and possible production of AF[11, 47]. The finding that the most contaminated samples were from the Eastern region of Rwanda is consistent with Cotty and Ramon [39] as hot, dry weather favours AF formation [48]. The infection by *Aspergillus,* the sole producer of AF, is higher when the temperature in association with drought increases (>25°C) [39]. Aflatoxins occur mostly in tropical and sub-tropical regions where high humidity and temperature are recorded [18, 49]. Pre-, peri- and post-harvest conditions and agricultural practices play critical roles in modulating the risk of mycotoxigenic fungal colonization and growth, as well as mycotoxin contamination[49, 50].

### Multi mycotoxins in soybean

The four parameters required to confirm a mycotoxin presence were not fulfilled for most of the mycotoxins analysed: OTA, AFB1, AFB2, AFG1, AFG2, DON, ZEN, FB1, FB2, T-2, HT-2, ROQ-C, NIV, NEO, FB3, AOH, AME, 3-ADON, 15-ADON, DAS and FUSX. The signal to noise ratio was <3 for all “legislation” mycotoxins in all samples. One subsample had a S/N ratio >3 for STERIG. Sterigmatocystine was at a level of 13 µg/kg (Kirehe sub-sample 2 in the Eastern agroecological zone). Sterigmatocystine is among mycotoxins, which do not have maximum levels set by EU [51, 52]. According to the EFSA’s Scientific Opinion on STERIG in food and feed, the acute oral toxicity of STERIG is relatively low [53].

While other studies have found that mycotoxin levels are generally low in soybean and its products globally, there are reports that contamination can still be a problem. In a study in Brazil, the reported average values were 0.5 µg/kg, 30 µg/kg and 57 µg/kg for AF, ZEN and FB, respectively [54]. Soy foods marketed in Germany, including whole beans, roasted soy nuts, flour and flakes, textured soy protein, tofu, protein isolate, infant formulas and fermented products (soy sauce) were analyzed for *Fusarium* toxins, and 24% of the soy foods were contaminated, mostly with DON and ZEN [55]. In a four-year surveillance for OTA and FB in retail foods in Japan, 13 soybeans samples out of 20 were contaminated with FB1 (mean, 4.5 µg/kg), where 3 samples out of 20 were contaminated with FB2 (mean, 4.3 µg/kg), and OTA was not detected [56]. The OTA contamination of Korean fermented soybean was not high (0.14 µg/kg) [57].

In Rwanda, no study was done on soybean contamination by mycotoxins. Aflatoxin contamination of maize can be a problem, similar to reports from other countries in the region. Maize in principal retail markets in Rwanda was found to be contaminated by aflatoxin (2-35% and 66-100% of samples were above the US and EU limits, respectively) with AF up to 26 µg/kg [36]. A three-year survey done on soybean in America, Europe, Asia and Oceania on the mycotoxins occurrence in feed proved that soybean was mostly contaminated by FB (range, 12 µg/kg – 2,966 µg/kg) [58]. These studies clearly show that soybean is less contaminated in comparison to cereals like maize. However, it is necessary to remain vigilant, and conduct periodical analysis of mycotoxin contamination in soybean food and feed. The results show relevance of identifying low risk, nutritious food (soybean) to policy makers in Rwanda.

## CONCLUSION

The results obtained in this study constitute baseline information on mycotoxins contamination in soybeans in Rwanda. According to the reported literature and findings of this investigation, Rwandese soybean is safe for human consumption because the soybean samples from Rwanda had acceptably low mycotoxin levels. Therefore, soybean can be promoted as a safe and nutritious food including for weaning, with good agricultural practices helping to ensure that contamination remains low. Given its importance as a major protein source in the human and animal diet, there is a need to promote the cultivation and consumption of soybean in Rwanda. However, there is need for educating farmers on mycotoxin contamination in food and feeds to ensure better standards to safeguard the health of the consumers regarding these fungal metabolites. A larger surveillance on these toxins should be done in the Eastern region, for a range of susceptible crops, as the weather is more conducive for mycotoxin production.
